# The Rhodesian Anthrax Epidemic: A Case of Biological Warfare?

**DOI:** 10.7759/cureus.85977

**Published:** 2025-06-14

**Authors:** Matthew D Turner

**Affiliations:** 1 Emergency Medicine, Penn State Health Milton S. Hershey Medical Center, Hershey, USA

**Keywords:** anthrax, biological warfare, cbw, chemical and biological warfare, epidemic, rhodesia, rhodesian bush war

## Abstract

The Rhodesian anthrax epidemic of 1978-1980 was one of the largest in recorded history. Occurring during the chaos of the Rhodesian Bush War, the anthrax epidemic led to the deaths of hundreds and caused significant economic devastation in what is now the country of Zimbabwe. To this day, the origins of this devastating outbreak remain highly controversial. However, in this article, we determine that the anthrax epidemic most likely occurred due to an endemic outbreak, exacerbated by the breakdown of services from the war, food scarcity, and a number of other unique wartime circumstances.

## Introduction and background

The Rhodesian Bush War

The colony of Southern Rhodesia, now known as Zimbabwe [1], was originally established in 1889 under the British South Africa Colony, a chartered entity under Cecil Rhodes, whom the colony was named after. In 1922, the territory switched to a self-governing colony under Great Britain [2]. 

The Rhodesian 1961 constitution ensured European dominance of the colony’s governance, despite the fact that the White population of 220,000 was only 6% of the entire population. As part of the decolonization process of the British Empire, London required that Rhodesia transition to majority rule before the colony could be granted independence, a process that had the support of the United Nations. Prime Minister Ian Smith refused these demands and declared Southern Rhodesia independent via the Unilateral Declaration of Independence (UDI) on November 11, 1965. In Resolution 216 of 1965, the United Nations formally condemned the UDI, and in its subsequent Resolution 217, it declared that, given the “usurpation of power by a racist settler minority,” it would refuse to recognize the former colony [3]. Strict international sanctions followed [2]. While the British continued to refer to the former colony as Southern Rhodesia, most of the world and the Rhodesians themselves referred to it as simply “Rhodesia” [1], which this paper will emulate for the purposes of simplicity.

The full scale of the Rhodesian Bush War is outside the scope of this paper. However, in light of Rhodesian independence and the Smith government’s refusal to grant majority rule to its native African population, a smoldering civil conflict soon developed, running from 1965 to 1979 [1]. It soon developed into a three-way conflict between the Rhodesian security forces and the Zimbabwe African National Liberation Army (ZANLA) and the Zimbabwe African People’s Revolutionary Army (ZIPRA), military wings of the Zimbabwe African National Union (ZANU) and the Zimbabwe African People’s Union (ZAPU), respectively. All three factions had differing objectives, and guerrillas from the ZANLA and ZIPA forces often came into direct conflict [4].

The war was punctuated by multiple atrocities and rapidly became more vicious and brutal over time. The Smith government became rapidly more racialized and authoritarian [2], and by 1976, it had abandoned all pretenses of routine judgment, creating special courts specifically designed for captured guerrillas. No records were kept of executions, although a 2002 United Nations report noted the discovery of a previously secret diesel-powered crematorium located near one of the state’s maximum-security prisons [4]. As the war progressed, the government passed harsher and harsher legislation, including mandatory death sentences for the throwing of petrol bombs and possession of illegal arms. These provisions drew international condemnation. In 1968, Queen Elizabeth II unsuccessfully tried to use a royal pardon to save three men on death row. Although Rhodesian authorities eventually walked back the mandatory death sentences, by 1975, the government declared that the names of executed prisoners would no longer be released, leading to hundreds of secretive executions [5].

The early stages of the war went well for Rhodesia. Rhodesia’s initial counterinsurgency operations were highly effective due to a small but well-funded and highly professional army, an effective intelligence network, and clandestine economic and military support from South Africa [6]. In the field, Rhodesian forces had an overwhelming tactical advantage and were judged as some of the best in the world at guerrilla and counter-insurgency operations [6].

Despite early successes, by 1978, it was obvious that Rhodesian forces were fighting a losing war. Manpower and resources were running critically low, and the country’s early successes at evading international sanctions were rapidly running dry, even as the number of guerrillas continued to grow [1]. As the state became increasingly desperate, it turned to more unconventional tactics, such as releasing large numbers of booby-trapped radios into the civilian community in an attempt to flush out guerrillas [4]. It was in this context that Rhodesia enacted one of the most brutal Chemical and Biological Warfare (CBW) programs ever seen.

Rhodesian CBW efforts

The use of chemical and biological weapons in warfare is as old as humanity itself [7]; for example, there is evidence of humans using poisoned arrows in the prehistoric age [8]. However, with the advent of the industrial age, the mass production of chemical agents, and humanity’s ever-improving ability to cultivate and deliver dangerous microorganisms, CBW agents have become exponentially more threatening [9]. While these agents have been used in recent wars, an international taboo has existed over their use for decades [1], with international agreements such as the 1975 Biological Weapons Convention forbidding their use [10].

As the security situation deteriorated, Dr. Robert Symington, a professor of anatomy at the University of Rhodesia’s medical school, proposed a new counterinsurgency strategy based upon CBW. The program was approved by the highest levels of the Rhodesian government, which closely followed the program’s development and deployment of chemical and biological weapons throughout the war [1].

Drawing from a number of medical and veterinary students from the University of Rhodesia, Symington allegedly began human experimentation as early as 1975 [1]. The CBW program experimented with a number of compounds, including warfarin, parathion, thallium, and *Vibrio cholerae* [1].

Despite a lack of resources and primarily relying upon commercially available agricultural products, the program was brutally effective. Dr. Symington was said by one officer to “have killed more terrorists than the Rhodesian Light Infantry” during certain months of the war [4]. The CBW program saw its greatest successes with the use of parathion, an organophosphate used in pesticides. In isolated locations, CBW employees soaked hundreds of articles of clothing in parathion solutions, afterwards drying them to eliminate the smell. The poisoned clothing was then distributed to guerrilla forces through local contacts who would directly donate it to guerrilla recruits, by contaminating discovered caches of guerrilla supplies, or by stocking stores that were in guerrilla-heavy areas and expected to be ransacked [1].

Other uses of CBW included the deliberate poisoning of foodstuffs with thallium and warfarin, as well as the deployment of cholera to contaminate water sources known to be frequented by guerrillas [1]. This use of cholera in warfare made Rhodesia the first nation to intentionally deploy biological weapons after the 1975 passage of the Biological Weapons Convention [10].

While exact numbers are impossible to obtain, the Rhodesian use of CBW killed hundreds to thousands of guerrillas. Up to 15% of casualties in the entire war may have been due to CBW [1]. Even with extremely limited resources, the small CBW program was judged as a success by Rhodesian forces [1].

Anthrax

Anthrax is endemic to the region [1]. *Bacillus anthracis* is a Gram-positive bacterium that may exist in the environment as a spore for decades. Typically ingested by grazing animals, it will replicate and eventually kill its host, with the meat and hide from the animal serving as potential contaminants for humans [11].

There are several distinct clinical manifestations of the disease in humans. The vast majority of human infections are due to cutaneous infections of anthrax, when spores are introduced through breaks in the skin. After one to 12 days, the patient will develop an edematous papule that will gradually evolve into a 1-2 centimeter vesicle, with significant local lymphadenopathy and fever. Within several weeks, the vesicle ulcerates into a black eschar, which eventually sloughs off [11]. With adequate treatment, cutaneous anthrax has a mortality rate of less than 1% [11].

The more severe clinical presentations are significantly rarer. Gastrointestinal anthrax is usually due to contaminated meat and has a mortality rate of approximately 25% to 60%. Patients may present with ulcerative lesions within their intestines, causing severe ascites that may lead to internal bleeding and intestinal perforation [11]. Inhalational anthrax is usually due to industrial outbreaks, typically in factories that process contaminated animal hides. With an extremely high mortality rate, these patients often go into septic shock, with bacteremia and meningitis present [11].

## Review

The anthrax outbreak

It was in this context that the largest confirmed anthrax epidemic in recorded history broke out in Rhodesia in 1978 [12]. From 1978 to the mid-1980s, the epidemic rapidly encompassed the country; reported human cases ranged from 10,738 to 17,068, with confirmed fatalities ranging from over 100 to over 200 [12]. However, human cases were almost certainly underreported; in many cases, patients either died at home or were unable to present to a medical facility due to the ongoing conflict [12]. The epidemic was rapid and explosive in its onset; in the prior year of 1977, there was not a single report of human or bovine anthrax reported in the entire country [13].

One of the most important aspects of Rhodesian society was the difference between Tribal Trust Lands (TTLs) and commercial farms. Over 60% of the country’s African population lived on TTLs, where they engaged in subsistence agriculture, in contrast to the European-owned commercial farms, which focused on the production of cash crops [1]. This segregationist policy between the poor farmland of the TTLs and the much more productive land of the commercial farms severely hindered the economic prospects of the local African community and was a source of significant tension [14]. While data are limited due to the breakdown of communications and veterinary services with the war, it appears that the epidemic originated with TTL cattle in mid-1978 in the Nkayi District of the Matabeleland North Province, located in the eastern half of Rhodesia. The first reported human case occurred on 24 November 1978, when a patient presented to a nearby rural hospital with a cutaneous anthrax lesion, which was determined by physicians to be the result of skinning and butchering an infected cow [1]. Over the next two years, thousands more human cases would be reported in this area, with anecdotal evidence suggesting that many patients had died before they were able to access medical treatment [1]. Within six months, the epidemic had spread to the bordering Que Que district [1]. From here, the disease had a very unusual progression across the region. Instead of spreading contiguously, contemporary observers noted that the local outbreaks “hopped” across bordering regions, with local outbreaks occurring as far as 135 kilometers from the initial epicenter [1].

By October 1979, it was obvious to authorities that the country was experiencing an unprecedented epidemic. The Zimbabwe-Rhodesian Department of Veterinary Services issued a health warning and reported that over 500 people had been treated for anthrax, with 21 fatalities [1], almost certainly a gross underestimate. Within three days of the report, the government abruptly announced that the anthrax had spread to another six TTLs [15]. Reported cases suddenly surged in late 1979 and early 1980, as the epidemic spread into TTLs encompassing nearly 120,000 square kilometers in Mashonaland Province [12]. While much of the disease spread contiguously toward the northeast, non-contiguous spread of the disease was again observed, with “hops” of 40-50 kilometers [12]. In his contemporary report on the epidemic, Dr. J.C.A. Davies noted the “rather puzzling” hops that the epidemic often displayed, often skipping over intervening areas of European-owned commercial agriculture [13]. By the fullest extent of the outbreak, 17% of the country was affected [1]. Medical facilities reported significant strain on their resources managing the epidemic, with multiple hospitals concerned about the lack of personal protective equipment [12,16]. The strain on the medical system continued into the early years of reconstruction after the war’s conclusion due to the closure of large numbers of rural hospitals [13].

In all known cases, bovine infection preceded human infection [12]. Within the TTLs, the agricultural and economic strain from the epidemic was immense. Some communities reported losing up to 90% of their cattle [1], and estimated numbers of cattle deaths ranged from 170,000 [12] to as high as 1,000,000 [1] out of an estimated 1977 TTL cattle population of 3,338,000 [1]. In contrast, the European-owned commercial ranches only reported 11 cases of bovine anthrax from 1978 to 1980 [1]. In addition to this, every reported anthrax case in humans was confined to rural African populations; not a single case of anthrax was reported in the country’s European population [1]. Despite initial concerns in hospitals, there were no reported cases of cross-contamination to healthcare workers [12].

Possible bioweapon release

Given the Rhodesian government’s well-documented CBW program and use of other agents, including warfarin, cholera, thallium, ricin, and widespread use of organophosphates such as parathion [1], the anthrax epidemic of 1978-1980 is suspicious. Dr. Meryl Nass, an American physician living in Rhodesia at the time, was among the first to propose that the anthrax outbreak was manmade in a 1992 paper [17], an assertion that has been supported by later authors [15,10]. For the purposes of this paper, the arguments made by Nass and later authors for a deliberate bioweapon release will be listed below.

The Scale of the Epidemic

With the exception of a possible outbreak in Haiti in 1770 [18], the Rhodesian anthrax epidemic of 1978-1980 was the largest in recorded history [1]. Since the end of the Bush War, Zimbabwe has experienced no anthrax outbreaks even approaching the sheer numbers of its colonial predecessor [1]. The rapid speed and volume of the outbreak were so vast that contemporary observers compared it to a 1979 outbreak of anthrax in the Soviet city of Sverdlovsk [12]. An outbreak that was later found to be accidental research from a Soviet biological weapons laboratory [19]. While anthrax was endemic to Rhodesia, Nass argues that the unusually large scale of the epidemic indicates it must have been an intentional release [1].

Geographic Spread of the Epidemic

Typical outbreaks of anthrax are limited to a small geographic area. Here, cattle will consume anthrax spores, and human cases of anthrax will develop from eating or handling infected bovine carcasses [1]. Previous small outbreaks of anthrax in Rhodesia had followed this model, which made it easy for officials to identify a point source of the epidemic [1]. However, the 1978-1980 anthrax epidemic spread across a vast geographic area, encompassing nearly 250,000 square kilometers at its height [12]. Officials were never able to identify a point source for the epidemic [1]. In addition to this, the geographic spread of the disease was also very unusual, as it was seen to “hop” over entire communities [13].

As part of the unusual spread of the disease, the anthrax epidemic was entirely limited to Rhodesia. No surrounding countries reported a single case of anthrax during this time span [1]. Other than humans and bovines, there were no other reported animal sources of the epidemic [12]. A possible wildlife source of the epidemic appears to be extremely unlikely; the first wildlife infection with anthrax in Zimbabwe was detected in 2004 [12], and wildlife infected with anthrax in that region remains rare to the present day [1].

Possible Targeting of African Communities

As part of the “hops” that the anthrax epidemic demonstrated, it appeared to preferentially target the local African community. All of the thousands of reported human cases were rural Africans; no White Rhodesians appear to have ever been impacted by the disease [1]. This extended to the cattle herds as well; while communal cattle on the TTLs were devastated, the European-owned commercial farms only reported 11 cases of bovine anthrax throughout the entire epidemic [1].

Clinical Presentations

The Rhodesian anthrax outbreak was also unusual in that all clinical presentations of anthrax, inhalational, gastrointestinal, and cutaneous, were present during the outbreak [13]. Severe cases of meningitis secondary to the disease were also detected [12]. In comparison, the vast majority of naturally occurring anthrax outbreaks are limited to cutaneous manifestations of the disease [20]. When it does occur, inhalational anthrax is typically associated with textile mills that use contaminated animal skins [21]. Interestingly, despite its large hide processing industry, Rhodesian factories had no significant outbreaks of inhalational anthrax; all cases of inhalational anthrax were limited to rural regions [1]. This may be because Rhodesian factories received their animal hides from the unaffected commercial farms [1].

Anecdotal Reports

Many rural inhabitants claimed to witness government airplanes dropping a mysterious “white powder” on pastures and cleared land that they believed directly led to the anthrax outbreak [22]. This may have been corroborated by an interview with a senior Rhodesian officer in 2000, reported on in a 2002 United Nations report, who stated that the Rhodesian Special Air Service was tasked with deploying anthrax from aircraft [4]. Guerrilla forces were also well aware of the Rhodesian government’s use of chemical weapons and suspected that security forces were attempting to do the same with biological weapons. Some believed that Rhodesian forces were deliberately infecting cattle with anthrax to poison the guerrillas that would later eat the meat [22]. Later on, many Zimbabwean officials, including Tim Stamps, the Minister of Health, remained convinced that the anthrax epidemic was deliberately spread by Rhodesian forces [15].

Anecdotal reports from Rhodesian forces also suggest the possible deliberate use of anthrax. In a 2002 article, former CIA intelligence officer Ian Martinez discussed reports of Rhodesian officers discussing the use of anthrax “in an experimental role in the Gutu, Chilimanzi, Masvingo, and Mberengwa areas…”. According to these officers, the anthrax was meant as a psyops campaign deliberately targeting tribesmen to suggest to locals that their cattle were sick and dying from diseases carried by infiltrating guerrillas [15]. This was part of a wider campaign “to alienate the local population from the insurgents” [15]. One of the commanders of the elite Selous Scouts reported that he was once ordered to distribute anthrax by his superiors but refused due to concerns about the danger it posed to his men [1]. A 2002 United Nations report determined that, at a minimum, Rhodesian forces had deposited anthrax within the Botswana border; however, this source was solely based upon an interview with a senior Rhodesian officer [4].

The Rhodesian CBW program also was reported to experiment with anthrax supplied from South Africa, initially in contaminating cigarettes with anthrax to kill specific targets [1]. At a bare minimum, it appears that the Rhodesian government was well aware of anthrax and had considered its potential as a biological weapon [1].

Deliberate Targeting of Cattle and Food Stocks in the War

As the war worsened and atrocities mounted, cattle grew to become a target for Rhodesian security forces. Civilians suspected of cooperating with the guerrillas were often punished via the burning of their households and the slaughter of local herds. While the government later attempted to tamp down on the needless killing of livestock, they were frequently a target in the war by frustrated security forces [6]. In one typical incident in September 1979, a Rhodesian task force, hot on the tracks of a guerrilla band, came across an elderly man tending to a small group of cattle. Angry and “with their blood up,” the security forces slaughtered the entire herd. The brutal strategy worked; the weeping owner pointed them in the direction of the fleeing guerrillas [6]. In this environment, where cattle were often the economic heart of African communities [12], it is possible that the anthrax epidemic may have been specifically targeted against these herds. In the local populations, cattle were an indicator of wealth; to lose one’s cattle was to lose one’s livelihood [15].

In addition to this, the Rhodesian security forces often targeted the food supplies of their enemies and their own population. In addition to regularly poisoning food with poisons such as thallium or warfarin, either in guerrilla camps or in stores and villages suspected of aiding guerrillas [1], the Rhodesian government instituted Operation Turkey in 1977. Operation Turkey strictly limited the food supply of the native population through several measures: European farmers were prohibited from supplying African laborers with more than one day of food supplies at a time, ration cards were distributed to the population [15], legal limitations were applied on the numbers of foodstuffs that stores could possess, and a strict crackdown on food transportation between different regions went into effect [1]. Checkpoints where locals were searched and excess “contraband” food destroyed became increasingly common [1]. The goal of this strict control of food supplies was aimed at both reducing the amount of local food available to guerrillas and increasing animosity between the local populations and guerrilla forces [15]. As Martinez argues, deliberately spreading anthrax would’ve been highly effective at reducing the food stocks of the local population [15], especially given the reliance on TTL cattle for sustenance in times of food insecurity [12].

Military Considerations

Anthrax has been used as a biological weapon since the First World War due to a number of factors, including its persistence in the environment, the ease of dissemination, and the fact that it may be attributed to a natural outbreak, ensuring plausible deniability. Germany deployed anthrax throughout Romania, Spain, France, Norway, Argentina, and the United States during World War I. The British Empire maintained a stockpile of over 5,000,000 anthrax cakes to be deployed via parachute over Germany in “Operation Vegetarian” during the Second World War. Multiple other nations have experimented with anthrax and maintained viable stockpiles [23].

Martinez points out that the use of anthrax would have aligned with several of the Rhodesian security forces’ other practices during the war. It would have further alienated local populations from guerrillas by suggesting that guerrillas were bringing in “foreign” diseases; it would have further reduced the country’s food supply consistent with Operation Turkey, and the economic devastation of the anthrax epidemic would have broken the morale of the rural population [15]. All of these accomplishments would have contributed immensely to the Rhodesian war effort.

Martinez also points out that the first case of human anthrax was reported in November 1978, the very peak of hostilities. While it was already obvious that the Rhodesian forces could not hold out indefinitely, an anthrax outbreak could have strengthened the European position in a settlement agreement by “illustrating that the Black population was enduring the worst consequences of the war and thus had the most to gain through a negotiated settlement” [15]. This would have been consistent with other Rhodesian behavior; towards the end of the war, the government became increasingly desperate for any victory that could improve its bargaining power [6].

A natural outbreak

The alternative explanation for the massive anthrax epidemic was still that it was a natural outbreak, exacerbated by the ongoing conflict. While the anthrax epidemic was unprecedented in its size and scope and had several unique presentations, much of this can be attributed to the unique wartime circumstances of Rhodesia [1]. Even taking into account the Rhodesian CBW program [1], it appears likely that the epidemic was natural in origin.

Breakdown of Veterinary Services

Cross argues that the country’s political instability played a massive role in the anthrax outbreak [1]. The war saw a complete breakdown of Rhodesia’s once-excellent veterinary system [12]. Control over parasites such as the infamous tsetse fly utterly collapsed as the government lost more and more access to rural and outlying regions, with the country’s chief veterinary officer bemoaning that the ongoing conflict had “put back Rhodesia’s battle against the tsetse fly 20 years” [24]. Other diseases, including foot-and-mouth disease, tick-borne diseases, rabies, and trypanosomiasis, exploded as well [25]. [12]. The problem was due to multiple factors. As the war progressed, veterinary services lost nearly all access to the rural areas of the TTLs [13]. This was due to both distrust [12] and a loss of support from the local population. By complying with government vaccination, TTL inhabitants were often afraid of being labeled as government collaborators [1]. Limited supplies and insurgent attacks further exacerbated the situation [12]. By 1979, only 1,500 out of the country’s 8,000 cattle dips, used for inoculation, were still in operation [26]. By 1977, the veterinary situation was so dire that even outside observers were well aware of the country’s deteriorating situation [27]. This is similar to other African countries that have experienced warfare and political instability, with an associated increase in diseases such as trypanosomiasis [28].

Anthrax was endemic to Rhodesia but had been well controlled for decades under one of the “most advanced and effective” veterinary programs on the continent [12]. Physicians were said to have an “almost cavalier” attitude toward the disease, which had previously posed no significant threat or concern [13]. From 1926 to 1977, the disease had only had 322 reported cases in humans, with only 20 fatalities in total [12]. As noted above, there was not a single report of bovine or human anthrax in the entire country in the year before the epidemic [13]. However, the ongoing collapse of the veterinary system also disrupted the cattle vaccination program immensely [1]. Even by 1977, a year before the outbreak of the epidemic, the impact on African-owned cattle was devastating; African farmers had gone from producing 70% of the country’s foodstuffs prior to the war to only 30% [26].

In one notable example, Nkai Rural Hospital was near the epicenter of the original epidemic in November 1978. Senior medical personnel at the hospital noted that this period marked the very peak of the war and that none of the local dip tanks were functional at the time, preventing any inoculation of cattle [13]. In addition to the widespread loss of preventive measures in the TTLs, the government lost any ability to respond to ongoing outbreaks due to the security situation [1]. Standard and previously effective procedures, such as burning carcasses, vaccinating surrounding livestock, prohibiting the movement of nearby livestock, and establishing quarantines, all became impossible as the Bush War spread across the countryside [1]. Many contemporary Rhodesians blamed the anthrax epidemic on the guerrillas’ “antidipping” campaigns [22]. It is also important to note that anthrax was not the only bovine disease to explode during this time; Zimbabwe officials have pointed out that rabies and tick-borne infections “escalated several 100-fold” contemporaneously with the anthrax outbreak [29], further supporting the veterinary services collapse theory.

In contrast, the European-owned commercial farms, designated as Vital Asset Ground by security forces [12], had only 11 reported cases of bovine anthrax throughout the epidemic [1]. Unlike the rural TTLs, these commercial farms retained access to bovine vaccination and excellent veterinary care throughout the war, likely explaining their resilience to the outbreak [1].

TTLs and Commercial Farms: Differences in Herding

In addition to their improved vaccination and access to veterinary care during the war, Davies pointed out several differences in agricultural techniques between the TTLs and the commercial farms. Rhodesian commercial farming employed a system called the “Savory System,” where groups of cattle were moved at regular intervals from paddock to paddock in a planned rotation. This minimized contact between various groups of cattle, offering another degree of protection against disease [30]. Commercial farms would also often pipe in water during feeding times [30], further reducing the risk of cross-contamination.

In contrast, the TTLs used communal farming areas, which inevitably led to significant mingling between herds, thus increasing the risk of disease. Watering points were more difficult to obtain, leading them to become more overcrowded. Davies theorized that this meant cattle in the terminal stages of anthrax infection would be far more likely to contaminate communal water sources through the discharge of anthrax bacilli [30]. Modern research supports this; anthrax spores appear to often collect in water storage areas, making watering points dangerous areas where animals may transmit the disease [31]. Already under severe duress by 1977 [26], it appears that the TTLs were exceptionally vulnerable to an anthrax outbreak.

Consumption of Infected Carcasses

European and African approaches to veterinary medicine in Rhodesia had been markedly different. In addition to the farming differences between the commercial farms and TTLs, Rhodesian veterinary medicine placed a strong emphasis on burning or burying bovine carcasses suspected of disease [1]. This had been a source of tension for the rural African population for decades, who felt that such practices invariably benefited the European-owned herds. In one prophetic complaint, uttered over 70 years before the anthrax epidemic, one farmer complained, “Whoever had heard of food being destroyed like this? If our cattle die well, we could eat them, but these people bury and burn them, and grain is scarce. They want us to die of famine.” [32]. Even before the anthrax epidemic, the country’s rural food supply was erratic; a 1976 study of farmworkers’ children found that 90% of the children under five years of age were malnourished [33].

This situation was only exacerbated with the breakdown of services from the Bush War and the government’s focus on strict caloric control of the rural population through decrees such as Operation Turkey [1]. By late 1979, the International Red Cross estimated that 13% to 29% of the population was experiencing malnutrition [33]. In much of Rhodesia’s rural regions, the massive increase in food scarcity meant that meat became a critical source of food [1]. As a result, consumption of anthrax-tainted meat became a common means of transmitting the disease. One observer noted that dead cattle “are almost always cut up and eaten by people before the predators have a chance.” In a typical rural area, a single carcass could be butchered and its meat sold to 20 families, potentially exposing over a hundred to anthrax consumption [1]. With the advent of Operation Turkey, black-market smuggling of meat between the various TTLs rapidly increased. Dr. Davies theorized that one of the reasons for the disease’s rapid spread was that the “meat of the carcasses infected by anthrax [was] being sold around to the public as meat slaughtered at home… so the disease spread rapidly as the meat was carried on scotch carts to distant places in the TTL…” [13]. Many infected patients did admit to eating “cooked meat of doubtful provenance” during the epidemic [34]. Not only would this black-market smuggling help explain the rapid spread of the disease, but it also explains the unusual “hops” seen by contemporary observers [1].

A recent outbreak of anthrax in Uganda, with 49 cases of human anthrax, 36 of which had gastrointestinal anthrax, was found to have originated from a single cow. Approximately 95 people ate the dead cow’s meat, which was later found to be contaminated with anthrax. Even ensuring that the meat was well-cooked or boiled for over 60 minutes was still associated with a significant chance of gastrointestinal anthrax [35]. This 2018 outbreak further demonstrates how rapidly a single case of bovine anthrax can contribute to widespread human infections. This case also bears a strong resemblance to a Rhodesian outbreak of anthrax in September 1979. In one case, a male presented with a cutaneous anthrax lesion after butchering and consuming a dead cow. Within 10 days, five of his close relatives were admitted to the same hospital for anthrax infection [13].

Cutaneous Spread: Cattle and Tabanids

The vast majority of cases of reported human anthrax in the epidemic were cutaneous lesions. In a contemporary account, Dr. Davies noted that patients were well aware of this, and a typical patient would present to the hospital, stating, “Cattle have died in our area, and now I have this sore.” [16]. An estimated 95% of cases in the epidemic were reported to be uncomplicated cutaneous anthrax [30].

One theory is that tabanids may have been another source of transmission during the Rhodesian anthrax epidemic, further contributing to the disease’s rapid spread. Tabanids, also known as horseflies, are large biting insects that leave deep, painful wounds. Native to the region, they often follow animal herds and have been proposed as a potential vector for anthrax infection [1]. Anecdotal reports noted that there seemed to be large increases in the fly population during the outbreak [36], and the first cases of the anthrax epidemic correlated with the start of seasonal tabanid activity [1].

Contemporary reports noted anecdotal evidence that many patients reported bites from tabanid flies, which often developed into cutaneous anthrax lesions [16]. Of note, pediatric patients, who were less likely to have direct contact with the carcass of an infected animal, were often found to be more likely to have a distribution of cutaneous lesions on the head, neck, and face, consistent with transmission via biting insects [16]. Even in the greater population, the majority of cutaneous anthrax lesions developed on the face, neck, hands, and forearms, rather than areas typically covered by cloth, such as the trunk [36].

The theory of arthropod transmission is supported by the current scientific literature. Multiple studies have demonstrated that insects are capable of transmitting lethal anthrax infections, whether through mechanical transmission through bites [37] or through contamination of vegetation that grazing animals then feed upon [38,39]. The World Health Organization has noted that cutaneous anthrax sores may develop by means of a fly bite [29]. This theory provides another mechanism for the rapid and natural spread of the Rhodesian anthrax epidemic.

Qualities of the Anthrax Epidemic

Another argument that the anthrax epidemic was natural in origin was the disease itself. Despite the sudden and significant strain that it placed on the country’s medical facilities, the anthrax outbreak displayed no special properties. Once patients reached the hospital, fatality rates were relatively low [12]. No cross-contamination of medical staff ever occurred, and there were no reports of significant antimicrobial resistance [12]. The majority of cases were successfully treated with penicillin, and while the country’s veterinary services floundered, the health services appear to have adapted relatively well. There was never any indication of a penicillin shortage [12].

Although all major types of anthrax were presented during the epidemic [1], it is important to note that approximately 95% of the cases seen by physicians were uncomplicated cutaneous anthrax [30]. Given the sheer size of the outbreak, as well as the widespread consumption of contaminated meat [1], it appears nearly inevitable that all major types of anthrax infection would have presented. In addition to this, the outbreak of anthrax in Sverdlovsk. Due to an accidental release of anthrax spores from a site studying biological warfare [19], it was associated with vastly higher rates of pulmonary anthrax than those seen in Rhodesia [34].

Failure to Spread to Other Nations

Despite being a landlocked country surrounded on all sides by other nations, none of Rhodesia’s neighbors reported a single case of anthrax during the epidemic [1]. Again, the unique wartime circumstances likely contributed to this. No wild animals appeared to have been infected with anthrax [1]. Human and cattle movement across the border was tightly regulated by armed patrols and extensive minefields [1]. Water and food supplies on either side of the Rhodesian border were a frequent target for the deployment of chemical weapons by Rhodesian forces, further limiting cross-border movement [1]. This cordon sanitaire likely prevented any international movement of anthrax.

Military Considerations

It is important to note that the Rhodesian CBW efforts were highly limited in both resources and expertise. One senior official described the program as “amateurish” [1]. The program only had rudimentary resources and a sparse handful of facilities, one of which was a laboratory built in Professor Symington’s personal residence [1]. None of the chemical weapons used were ever produced by the program; instead, it relied upon cheap, commercially available materials [22]. Protective equipment was so limited that Symington himself had to be hospitalized from an accidental exposure to one of the program’s organophosphates in 1979 [1]. In this context, it appears highly unlikely that any large-scale production of anthrax, on the scale necessary to produce the 1978-1980 epidemic, could have been accomplished within Rhodesia [1]. Even with the support of the sympathetic South African regime, which performed experiments with CBW weapons under Project Coast, there is no evidence to indicate any significant transfer of anthrax during the Rhodesian epidemic [1]. As Cross points out, intentional contamination of such a large area in Rhodesia “would have required a very large amount of spores spread over a wide area,” on a delivery and production scale far outside Rhodesia’s capabilities [1].

The anthrax epidemic also did not appear to significantly support Rhodesian military or political objectives. It is also important to note that the eastern regions of Rhodesia were the most thoroughly dominated by insurgent forces during the time of the epidemic, while the epidemic largely struck the central and western provinces [22]; from a strategic standpoint, a biological weapon distributed in this manner would be highly counterproductive. Anthrax was reportedly discussed by the Rhodesian National Joint Operations Center (NATJOC), but it was rejected due to the risk of spreading to the European-owned cattle herds. Beef was a major export for the embattled nation, and any threat to one of the few sources of income to the government was not worth any potential benefits in the minds of military planners [1]. Contemporary observers noted that the anthrax epidemic was an “economic disaster” [34], the last thing that an unrecognized country under severe international economic sanctions [1] would want.

Absence of Physical Evidence

Over the past 45 years, there has been no physical evidence to support the biological weapon theory. No production or storage facilities of anthrax have ever been identified, and no physical evidence of intentional dissemination, whether spore-infected cattle feed or anthrax cakes, has ever come to light [1].

Comparison with the 1974 Outbreak

Finally, comparisons with the 1978-1980 epidemic should be made with the 1974 outbreak of anthrax in Rhodesia.

In April 1974, the country experienced a limited outbreak of anthrax in the Mondoro TTL. A large and sudden number of deaths of cattle in the area were reported to the senior animal health inspector. The local veterinary services immediately initiated a vaccination campaign for the local populations of cattle, donkeys, sheep, and goats, limiting any further spread among the animal population. Only six human cases of anthrax resulted, all of which were associated with the skinning and butchering of the infected animal carcasses. All six cases demonstrated cutaneous anthrax lesions; two of the patients presented to medical facilities with meningeal signs and died within several hours of presentation. The remainder of the patients made a full recovery [40].

In this small case, several of the factors that contributed to the 1978-1980 epidemic are readily apparent: a robust veterinary response that would soon be crippled by the war [1], handling of infected carcasses, and consumption of contaminated meat. Had this naturally occurring outbreak occurred during the chaotic collapse of the country’s veterinary and security services in the later years of the war, it is possible it may have caused an epidemic of its own.

## Conclusions

In conclusion, while the Rhodesian anthrax epidemic of 1978-1980 displays many unique characteristics, these can be attributed to the unique wartime circumstances of the country. While the Rhodesian regime did experiment with CBW, the anthrax epidemic appears to have been a naturally occurring outbreak worsened by a collapse of civil services, severe food shortages, and spread by arthropods. The outbreak was economically devastating and, unlike other CBW efforts, would have served no wartime objectives for the embattled regime. There is no physical evidence to support any bioweapon release, with any evidence to the contrary being anecdotal in nature. It appears highly unlikely that there was anything intentional about the largest confirmed anthrax outbreak in recorded history.
